# Towards a Structural Mechanism for Sister Chromatid Cohesion Establishment at the Eukaryotic Replication Fork

**DOI:** 10.3390/biology10060466

**Published:** 2021-05-26

**Authors:** Sarah S. Henrikus, Alessandro Costa

**Affiliations:** Macromolecular Machines Laboratory, The Francis Crick Institute, London NW1 1AT, UK; sarah.henrikus@crick.ac.uk

**Keywords:** DNA replication, sister chromatid cohesion establishment, cohesin, replisome, microscopy, cryo-EM

## Abstract

**Simple Summary:**

Cohesion establishment between sister chromatids is essential for cell proliferation. We discuss in this review key observations that link sister chromatid cohesion establishment with DNA replication. Genetic, biochemical, and microscopy studies are critical to uncover that establishment factors associate with the replication machinery. Structural studies are starting to build a framework to explain how the replication machinery traverses cohesin and thereby transitions from one parental duplex DNA to two duplicated DNA filaments behind the fork. Sister chromatid cohesion establishment employs two pathways: one converts cohesin from parental to duplicated DNA, and the other pathway loads cohesin from the nucleoplasm behind the replication fork. We discuss how cryo-EM, combined with single-molecule imaging, could help explain cohesion establishment by discriminating between possible scenarios for cohesin bypass by the replication machinery.

**Abstract:**

Cohesion between replicated chromosomes is essential for chromatin dynamics and equal segregation of duplicated genetic material. In the G1 phase, the ring-shaped cohesin complex is loaded onto duplex DNA, enriching at replication start sites, or “origins”. During the same phase of the cell cycle, and also at the origin sites, two MCM helicases are loaded as symmetric double hexamers around duplex DNA. During the S phase, and through the action of replication factors, cohesin switches from encircling one parental duplex DNA to topologically enclosing the two duplicated DNA filaments, which are known as sister chromatids. Despite its vital importance, the structural mechanism leading to sister chromatid cohesion establishment at the replication fork is mostly elusive. Here we review the current understanding of the molecular interactions between the replication machinery and cohesin, which support sister chromatid cohesion establishment and cohesin function. In particular, we discuss how cryo-EM is shedding light on the mechanisms of DNA replication and cohesin loading processes. We further expound how frontier cryo-EM approaches, combined with biochemistry and single-molecule fluorescence assays, can lead to understanding the molecular basis of sister chromatid cohesion establishment at the replication fork.

## 1. DNA Replication and Cohesin Function

Several molecular processes cooperate to maintain chromosome stability. Faithful DNA replication and repair produce an accurate copy of the genome. Equal segregation of duplicated chromosomes then ensures that each daughter cell inherits one single copy of the genomic content.

**Figure 1:**
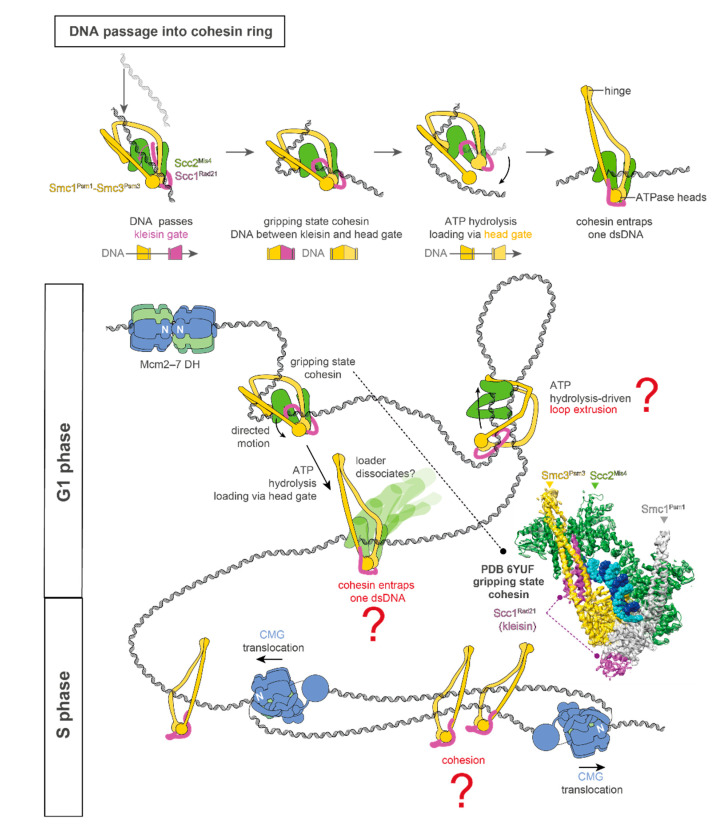
CMG formation and cohesin function in the G1 and S phases. At the top, model for DNA entrapment by cohesin. DNA is threaded through the open kleisin gate. In the ATP-stabilised “gripping” state, DNA is trapped between the kleisin and ATPase gates, while a DNA loop forms between the z-shaped cohesin loader (in green) and the cohesin hinge. ATP hydrolysis leads to the opening of the ATPase gate and ejection of the gripped DNA segment. As a result, the looped DNA remains entrapped within the cohesin ring. At the bottom, cohesin loading and loop extrusion at the origin of replication. During the G1 phase of the cell cycle, two MCM rings are loaded in a sequential manner, forming one DH on duplex DNA. Cohesin loading transitions via an ATP-binding-dependent intermediate, dubbed gripping state (cryo-EM structure is shown on the right). Following ATP hydrolysis, if DNA is directed through the ATPase head gate, cohesin could entrap duplex DNA within the Smc ring on the path to cohesion establishment. Alternatively, if DNA does not enter the cohesin ring, ATP hydrolysis may power loop extrusion. In the S phase, the CMG helicase is assembled, and translocation is induced with the N-terminal domain of MCM first. Cohesin transitions from parental duplex DNA to two duplicated sister chromatids.

In the G1 phase of the eukaryotic cell cycle, the origin recognition complex (ORC), together with Cdc6, loads two hexameric ring-shaped MCM helicases around double-stranded DNA (dsDNA) [1,2,3,4,5] (Figure 1). During this process, the loading factor Cdt1 renders MCM competent for origin recruitment by stabilising an open DNA gate in the helicase ring [2,3,6,7]. In a sequential, concerted mechanism [1], which requires ATP hydrolysis by MCM [8,9], two loaded MCM particles engage to form a head-to-head (N-to-N) double hexamer (DH) [2,10], which “licences” origins for replication [11].

Cohesin is a multimeric, flexible, ringlike protein complex that belongs to the structural maintenance of chromosome (SMC) proteins. It is initially loaded onto dsDNA at origin sites and plays a major role in organising duplicated chromosomes for equal segregation [12,13,14]. At two opposed ends of the cohesin ring, formed by subunits Smc1^Psm1^-Smc3^Psm3^ (*Saccharomyces cerevisiae* and *pombe* nomenclature), the ATPase heads and the “hinge” domain form two distinct heterodimerisation interfaces. These interfaces are separated by long coiled-coil domains, which can bend and support the folding of the cohesin ring in a collapsed state [15,16,17]. The ATPase heads are bridged by a kleisin subunit (Scc1^Rad21^), respectively forming two distinct gates for DNA entry, which open and close at different stages during the cohesin loading reaction [18]. The positively charged hinge lumen is critical for both nontopological and topological DNA association [19]. While it is debated whether a hinge gate is operated during cohesin loading onto duplex DNA [16,19,20,21], it is widely accepted that the loader complex named Scc2^Mis4^/Scc4^Ssl3^ (or human NIPBL/Mau2) plays a key role in this process by modulating both the Scc1–kleisin and the ATPase gates [22,23,24,25,26]. In fact, three recent studies combining biochemistry and cryo-EM on human, *S. pombe*, and *S. cerevisiae* proteins detail a structural framework for cohesin loading onto dsDNA. The three studies describe the structure of an ATP-binding-dependent DNA loading intermediate of cohesin, dubbed “gripping state” (Figure 1). In this state, duplex DNA is trapped in between the Scc2^Mis4^ loader and the ATPase heads and the Scc1–kleisin gate. Crosslinking/mass spectrometry and biochemical experiments on *S. pombe* proteins led to the establishment of the order of events that result in cohesin loading. The kleisin gate first opens upon ATP binding, allowing DNA passage, then closes as the loader clamps against the ATPase gate. Hydrolysis of ATP then leads to the opening of the ATPase gate, which completes DNA entry. If, however, the kleisin gate is not traversed en route to the gripping state formation, topological loading is not achieved upon ATP hydrolysis, possibly causing a DNA loop to be extruded by cohesin [16] (Figure 1). During loop extrusion, cohesin gradually grows and enlarges a DNA loop by reeling in DNA in an ATP-dependent manner [27,28]. In the human and *pombe* structures, cohesin was observed in a collapsed configuration with the hinge packed against the Scc3 subunit, a HEAT protein that binds duplex DNA and supports loading [16,17]. In the *S. cerevisiae* structure, cohesin was observed in an extended configuration, with the DNA trapped inside the Smc ring [21]. Future work is needed to establish whether this difference is due to differences in the subunit composition of the cohesin complex (the *S. cerevisiae* preparation lacked Scc3), or rather due to the different DNA substrate used. Other factors modulate the unloading of cohesin from DNA. These include Pds5, which promotes dissociation of cohesin from DNA if topological loading has not been achieved [29]. Similarly, Wapl works as a cohesin release factor that is counteracted by Scc2 reloading cohesin [30]. Structures of cohesin bound to Pds5 and Wapl will greatly increase our understanding of the cohesin unloading process.

An increasing body of evidence indicates that the Dbf4-dependent kinase (DDK), which phosphorylates loaded MCM double hexamers, hence dictating the G1/S cell cycle transition, also has a role in cohesin loading [31,32]. For example, in *Xenopus laevis* egg extracts, DDK is required for cohesin recruitment to chromatin, targeting sites near pre-replication complexes [33]. Likewise, in human cells, cohesin loading onto DNA requires DDK and the MCM helicase in the early S phase [34], though independently of helicase activation. Furthermore, budding yeast DDK phosphorylates the Ctf19 kinetochore protein, which in turn recruits Scc2–Scc4, and thus cohesin, to centromeres [35].

Upon switch into the S phase, phosphorylation events by the DDK and CDK kinases promote the recruitment of a set of firing factors onto DNA-loaded MCM double hexamers, causing a structural transition that facilitates origin melting on the path to replication fork establishment (Figure 1). Here, a set of CDK-phosphorylated firing factors recruits Cdc45 and GINS to DDK-phosphorylated MCMs. As a result, the Cdc45–MCM–GINS (CMG) holohelicase is formed in a process that causes dsDNA untwisting in preparation for origin opening [36,37]. In a subsequent step, Mcm10 promotes the transition from a dsDNA-interacting to a single-stranded-DNA-(ssDNA)-interacting form of the CMG, involving the ejection of the lagging-strand template from the central channel of the MCM ring [36,38,39,40]. At the same time, the ATPase-powered DNA unwinding function of the MCM AAA+ motor in CMG is activated, causing two N-to-N helicase particles to cross their paths, which in turn leads to opening a replication bubble, so that the advancing edge of the helicase becomes the N-terminal MCM face [2,36,41,42]. Once the replication fork is established, an N-terminal MCM loop splits the DNA strands at the fork nexus [43,44] so that only the leading strand template is retained inside the MCM central channel [39,45,46,47], and is spooled towards the C-terminal end of the helicase, via an ATPase-powered substrate rotational movement around the helicase ring [48]. At the rear face of the CMG helicase, the leading-strand DNA polymerase Pol epsilon (made of Pol2, Dpb2, Dpb3, and Dpb4) interacts with MCM and GINS, poised to capture the leading-strand template as it emerges from the MCM channel of the CMG [43,49,50,51,52,53,54,55]. At the leading edge of the helicase, GINS further engages with Ctf4, a homotrimeric replisome-organising factor, via the conserved Ctf4-interacting peptide (CIP) motif found in the Sld5 subunit of GINS [51,56]. Due to the homotrimeric nature of Ctf4, which is based on the β-propeller domain in the carboxy-terminal half of the protein, Ctf4 bound to CMG can further recruit two proteins bearing CIP motifs to the front of the advancing helicase. This CIP mechanism is employed by other replication factors, including Pol alpha, which primes Okazaki fragments on the lagging strand [53,54,56], and Dna2, a nuclease/helicase involved in Okazaki fragment processing [57]. Despite being a nexus between multiple replication factors, Ctf4 is dispensable for naked DNA replication in vitro [41], while it has been shown to play a role in parental histone recycling onto lagging-strand DNA, together with Pol alpha and Mcm2 [58]. Sister chromatid cohesion factors also employ a CIP mechanism to engage Ctf4 at the replication fork, as described below.

**Figure 2:**
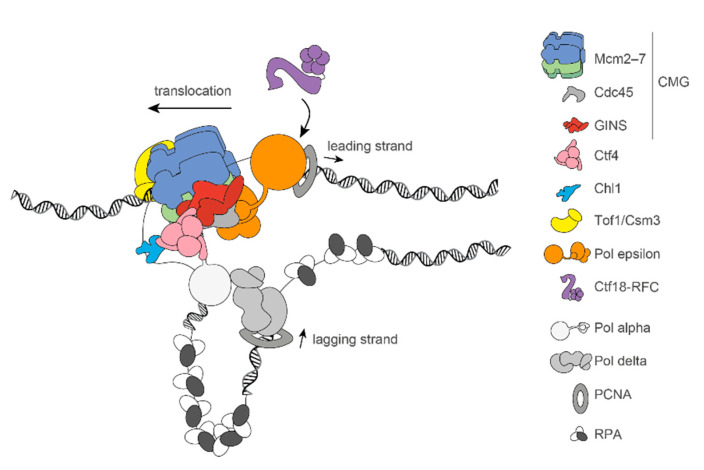
Architecture of the advancing replication machinery and positioning of cohesion establishment factors. CMG helicase (Mcm2–7 (blue-green), Cdc45 (grey), and GINS (red)) translocate N-terminal first. At the front of the helicase, Ctf4 homotrimer (pink) associates with the CIP box motif in GINS. Chl1 helicase (light blue) and Pol alpha (white) also bind to Ctf4 based on their CIP box motif. Pol alpha primes the lagging strand; RPA (black-white) associates with ssDNA between Okazaki fragments. The PCNA sliding clamp (dark-grey) supports discontinuous lagging-strand synthesis by Pol delta (light grey) at 3′ end of the primer, producing Okazaki fragments. At the rear of the replication machinery, Pol epsilon (orange) is engaged by PCNA, synthesising the lagging strand continuously. Ctf18–RFC (purple), an alternative clamp loader, interacts with Pol epsilon.

Sister chromatid cohesion is established following DNA replication initiation when cohesin transitions from entrapping parental DNA in front of the helicase to duplicated DNA behind the helicase. Two mechanisms have been proposed to describe the process of cohesion establishment (Figure 1 and Figure 2). The first widely accepted model pictures one cohesin ring transitioning from enclosing one parental duplex DNA to two duplicated duplex DNAs behind the replication machinery. This entrapment of two DNA molecules is called the ring model [59,60,61]. Several lines of evidence indicate that cohesin is likely to first enclose dsDNA from leading-strand synthesis and ssDNA from discontinuous lagging-strand synthesis [19,62,63]. ssDNA is then converted to duplex DNA by lagging-strand DNA polymerases, establishing sister chromatid cohesion. According to the second model, on the other hand, the linkage between sister chromatids is thereby established through cohesin–cohesin interlocking. Two cohesin molecules that topologically entrap individual DNA filaments behind the replisome could possibly link two DNA molecules [64]. In support of this model, endogenous wild-type *scc1* can compensate for the *scc1V137K* mutant, which has a defect in DNA–DNA tethering [19,65]. Overall, both DNA tethering models involve the topological binding of sister chromatids behind the replication fork.

Replisomal factors promote cohesion between sister chromatids by linking the cohesion establishment to the replication machinery. In support of this notion, immunoprecipitation from cells synchronised in the S phase led to the identification of a complex of Mcm2–7 with cohesin subunits Smc1, Smc3, and Rad21 [66]. Recent advances led to the description of how this interaction might be mediated, revealing that different sets of cohesion establishment factors are poised either at the front of or behind the advancing helicase (Figure 2). Ctf4 itself, which we introduced as a replisome-organising factor mapping on the leading edge of the advancing replisome, is also known to facilitate cohesion, suggesting that cohesion establishment factors might employ the CIP mechanism to engage Ctf4 at the replisome [53,54,56,67,68,69]. One of these factors is Chl1, a DNA helicase that functions in replication stress response and has a structural role in supporting cohesion establishment. Indeed, a Chl1 variant carrying mutations in the CIP motif has a cohesion establishment defect similar to a Ctf4 deletion, supporting the notion that Chl1 provides a direct physical link between the replication machinery and cohesin [70]. Chl1 is further required for the recruitment of the cohesin loader subunit Scc2 to chromatin during the S phase [71]. Predicted to be located behind the replication machinery, the Ctf18–Dcc1–Ctf8–RFC complex, an alternative loader of the PCNA processivity clamp, plays an essential role for cohesion between sister centromeres during meiosis [69]. While it is established that Ctf18–RFC directly engages the replisome by contacting the catalytic domain of Pol epsilon [72,73], its direct interaction with the cohesin complex is debated. In fact, reports of a complex containing Ctf18–RFC and cohesin subunits Smc1 and Scc1 kleisin are based on co-immunoprecipitation assays, which do not discriminate between direct and indirect binding [74]. Also interacting with processivity factors, Eco1 lysine acetyltransferase has a crucial role in cohesion establishment [75]. Consistent with acetylation taking place at the replication fork, Eco1 localises to replisomal sites in the S phase, and its interaction with the PCNA sliding clamp is crucial for cohesion and cell viability [76,77,78]. Yeast Eco1 (like human ortholog ESCO1) acetylates Smc3 at two conserved lysine residues, establishing topologically closed cohesin rings [29,79,80,81]. Acetylation of these lysines takes place sequentially, first, K112, then K113 [82], and counteracts the Wapl cohesin release factor, which mediates cohesin release from DNA [19,75,83,84,85,86,87]. Conversely, in the S phase, Wapl-independent release is inactivated through phosphorylation by Clb1/Cdk1 [30]. Recent cryo-EM work indicates that the two acetyl-acceptor lysines function as a signalling node [16], interacting with DNA and the Scc2^Mis4^ loader, respectively, explaining how these elements function in DNA-stimulated ATP hydrolysis by the Smc heads. Together, these cohesion establishment factors constitute a complex interplay on the path to sister chromatid pairing in the S phase.

At the start of the M phase, after pairing sister chromatids, loosely coiled chromatin condenses, and distinct chromosomes become visible [88,89]. When progressing through the M phase, double-strand break repair processes increasingly choose the homologous template over the sister chromatid in a process that involves cohesion and meiosis-specific axis components [90,91,92]. After aligning condensed chromosomes on the metaphase plate, midway between cellular poles, at the metaphase-to-anaphase transition, protease separin cleaves the Scc1 kleisin subunit [93]. Reversing cohesin’s topological entrapment of DNA molecules eventually allows the separation of sister chromatids in anaphase prior to cell division.

## 2. Coordination between DNA Replication and Sister Chromatid Cohesion Establishment

In the previous paragraph, we described how replisome-interacting factors facilitate cohesin activity in pairing and holding together duplicated DNA strands. At the same time, cohesin has an architectural role for DNA replication along chromatin loops [66]. Several studies have started to shed light on the process of sister chromatid cohesion establishment at the replication fork at the architectural and molecular levels.

Chromosome architecture strongly affects cellular processes such as DNA replication. During the S phase of the cell cycle, CMG helicases are formed at multiple neighbouring replication origins [94,95]. It has been proposed that these replication clusters are located in close proximity to each other with loops formed at interorigin DNA regions [54,96]. By examining DNA halo preparations of HeLa cells, cohesin downregulation was observed to cause an increase in fluorescent DNA halo, suggestive of increased loop sizes [66]. DNA loop size correlates with replicon length, and cohesin downregulation further negatively impacts DNA replication. When using signal from EdU incorporation as a proxy for replication foci, replication foci are observed to display reduced signal in cohesin-depleted cells, although the number of replication foci per cell is not affected [66]. Given that CMG protein expression levels are unaffected by cohesin depletion, this replication inhibition is not caused by changes in gene expression. While cohesin downregulation impedes DNA replication, MCM depletion does not affect cohesin occupancy along chromosomes. Conversely, in cells, increasing replication fork speed is detected upon cohesin acetylation and, as such, sister chromatid establishment [97]. Together, these observations suggest that cohesin’s nucleoskeleton supports efficient DNA replication. Alteration in the efficiency of DNA replication and related cellular processes may lead to DNA damage accumulation and thus to cohesin-related developmental disorders, collectively termed cohesinopathies [98].

**Figure 3:**
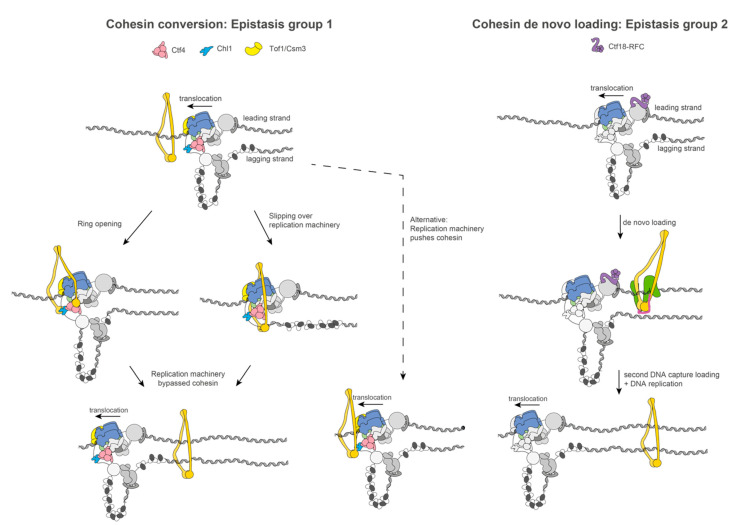
Two pathways for cohesion establishment: cohesin conversion and cohesin de novo loading. Cohesin loaded on parental duplex DNA transitions to two duplicated DNA molecules. According to the cohesin conversion model, this transition may involve the Smc ring opening or the cohesin ring slipping over the replicative machinery. This cohesin conversion pathway has been described to utilise the Ctf4 adapter protein (pink), Chl1 helicase (light blue), and Tof1/Csm3 fork protection factor (yellow). Alternatively, cohesin may be pushed by the replication machinery towards sites of replication termination. The second pathway, namely, cohesin de novo loading, depicts new cohesin molecules to be loaded behind the replicative machinery using nucleoplasmic cohesin molecules, which may originate from the cohesin dissociation of parental duplex DNA. This pathway utilises Ctf18–RFC (purple), which is located towards the rear of the replication machinery.

Single-molecule imaging and biochemical data revealed that sister chromatid establishment at the replication fork utilises two distinct pathways, known as Scc2-independent cohesion conversion and Scc2-dependent de novo loading. Single-molecule imaging of NRK cell clones indicated that the average residence time of cohesin on chromatin is 25 min [99]. In G1, ~40% of cohesin molecules are chromatin bound, while after the S phase, the bound fraction amounts to ~60% of cohesin molecules. This increase in chromatin-bound cohesins is likely caused by changes in the rates of cohesin loading and unloading. Cohesin accumulation on chromatin during the S phase suggests that some cohesin molecules may be bypassed by the replisome, and some cohesin molecules may be newly loaded behind the replication fork. Consistent with this notion, yeast genetic studies have uncovered parallel cohesion establishment pathways underlying two epistasis groups [100]. The first group consists of Ctf4, Chl1, and Csm3/Tof1, while the second group includes Mrc1 and CTF18-RFC. Depletion of two proteins, one from each epistasis group, leads to lethality or sickness and cohesion defects [100,101]. Based on these findings, sister chromatid establishment at the replication fork occurs via two mechanisms, namely, the Scc2-independent conversion of chromosome-associated cohesin molecules from one parental duplex to two duplicated DNA filaments and the de novo loading of cohesin onto duplicated DNA [101]. Each mechanism requires a different set of replisome-associated proteins that map on different sites of the replication machinery (Figure 3).

Scc2-independent cohesin conversion employs Tof1/Csm3, Ctf4, and Chl1, while Scc2-dependent de novo loading requires the Ctf18–RFC complex. As previously described, Chl1 is understood to engage Ctf4 at the front of the helicase [70]. Likewise, Tof1, a member of the Csm3–Tof1–Mrc1 fork protection complex, belongs to the same cohesin epistasis group as Chl1–Ctf4 [100,101] and is positioned towards the front of the helicase, and would be sandwiching the incoming parental duplex DNA, together with Ctf4 [43,48]. Csm3/Tof1 grips duplex DNA at the front of the helicase and may have a role in feeding the lagging-strand template towards the Ctf4-tethered Pol alpha, where Chl1 could also be found at the same time. Genetic and biochemical data support the notion that cohesin conversion and lagging-strand synthesis are intertwined processes. For example, one study identified an interaction between Okazaki flap endonuclease Fen1 and Eco1/Ctf7 acetyltransferase as well as Chl1 helicase [62]. Further, using the in vitro reconstituted cohesin loading system [24,26], a link was uncovered between DNA replication and DNA–DNA tethering, leading to the proposal of a three-step model for cohesin conversion. According to this model, (i) cohesin embraces one dsDNA molecule; (ii) second ssDNA is captured, resulting in a relatively labile interaction; and (iii) DNA synthesis establishes stable dsDNA–dsDNA entrapment by cohesin. In the process of second-ssDNA capture assayed in vitro, RPA has an inhibitory effect, which is partially abrogated when using an RPA variant, harbouring a point mutation in the ssDNA-binding OB fold of Rfa1 (*rfaG77E*) [102]. In agreement with this observation, studies in cells indicate that RPA overexpression causes strong sister chromatid cohesion defects. The same phenotype is however also observed for the temperature-sensitive RPA mutant, *rfaG77E*, which in vitro promotes cohesion. This discrepancy appears to relate to the two discrete cohesion establishment pathways. Indeed, cohesion defects are reduced in *rfaG77E* cells lacking Ctf18 of the de novo cohesin loading epistasis group. In Scc2-dependent de novo loading, Pol epsilon recruits the Ctf18–RFC sliding clamp loader through an interaction between Ctf18-1-8 and the catalytic domain of Pol epsilon behind the advancing CMG helicase [72,73]. Reloading of cohesin behind the fork may also involve one cohesin to tether two duplex DNA molecules independently of lagging-strand synthesis, which has previously been observed using optical tweezer imaging on cohesin loading reactions [61]. In summary, cohesion establishment is a versatile process where cohesin molecules can be reshuffled from the leading edge of the replication fork to the back of the helicase, but where cohesin can also be loaded from the nucleoplasm to duplicated DNA filaments.

Cohesion establishment is a highly dynamic process. At the global level of the human cell, replication clusters are organised by cohesin [66]; however, during the S phase, when the replisome encounters cohesins on parental duplex DNA, cohesin shows limited exchange dynamics in the absence of the Wapl cohesin release factor [103]. Wapl’s absence has no effects on sister chromatid cohesion establishment; rather, it eliminates exchange dynamics due to its cohesin unloading activity, which occurs independently of replisome encounter, as also observed in fission yeast [104]. While cohesin dynamically associates with and dissociates from chromosomes in the G1 phase, in a process that depends on *wapl*, during the S phase, it becomes less mobile, thus showing slowed diffusion. Cells treated with hydroxyurea (HU), to stall replication by depleting the nucleotide pool, still show cohesin stabilisation at both unreplicated and replicated origin sites [104]. This implies uncoupling between DNA replication and slowed cohesin dynamics, indicating that slowed dynamics do not occur due to cohesion establishment. In addition, Eco1^Eso1^ is not required for limiting cohesin dynamics in HU-treated cells. Together, these observations suggest that the cohesin dynamics are subject to factors that control association/dissociation to/from chromosomes and might be a prerequisite for cohesion establishment.

Single-molecule approaches are proving to be valuable to the study of cohesin function and dynamics. For example, using single-molecule fluorescence, cohesin molecules have been captured in the act of extruding DNA loops [27,28] or tethering two parallel, linear DNA segments in a process that requires one single-loading event [61]. Long lifetimes could be measured at the single-molecule level for cohesin on DNA, and ring mobility could be observed [105] with a diffusion coefficient that is reduced with lower ionic strength buffers, consistent with the electrostatic interactions between protein and DNA observed for several SMC complexes [16,17,21,106]. Although the structure of fully loaded cohesin on DNA is yet to be determined, single-molecule approaches reveal that the cohesin ring can pass DNA roadblocks of 10.6 but not 19.5 nm diameter [105]. A recent work further showed that loaded MCM DHs, which measure ~15 nm in width and ~20 nm in length, are semipermeable roadblocks for cohesin, only allowing ~20% of cohesin–DH encounters to resolve in cohesin passing DHs [107]. This implies that DHs, which are loaded onto replication origins together with cohesin in G1, can function to partially limit cohesin diffusion away from replication start sites. In solution under less controlled conditions, however, cohesin loading leads to cluster formation on DNA, promoting biomolecular condensations as observed by atomic force microscopy [108]. A very recent study discovered bridging-induced phase separation when working with DNA molecules that exceed 3 kb [108]. In vivo observations confirm that a subpopulation of cohesin molecules appears to associate with chromatin in a separated phase, consistent with previous observations of cohesin clustering observed in vivo [109,110,111]. Future work is required to identify any factor that might dissolve cohesin clusters to allow cellular processes such as DNA replication to take place.

## 3. Conclusions and Future Perspectives

Crystallographic, cryo-EM, and single-molecule fluorescence studies led to the description of the architecture and dynamics of cohesin, informing its function. The loading of cohesin onto DNA involves the formation of the ATP-binding-dependent gripping state intermediate where DNA is entrapped between Mis4 loader and ATPase heads [16,17,21]. Full topological loading requires ATP hydrolysis by Smc1–3; however, the structure of DNA-loaded cohesin still needs to be determined. Likewise, cohesin modulators such as Psd5 and Wapl affect cohesin engagement with DNA as they can favour cohesin unloading from chromosomes. The structural basis of how these factors affect DNA entrapment by cohesin may underlie an interaction with the Smc1/3 ATPase heads, which needs to be determined. Cohesin further executes fundamental DNA transactions, ranging from loop extrusion, where DNA loops are nucleated and enlarged in an ATP-hydrolysis-dependent manner [27,28], to entrapment of two DNA molecules [61,63] essential for cohesion establishment. Both functionalities appear to require one cohesin molecule to bind two DNA segments, either due to loop formation or due to entrapment of two separate DNA molecules; it however remains unknown whether both functionalities employ the same DNA-binding elements in cohesin. Another question relates to the conservation of the loading mechanism for other SMC proteins. Do condensin and Smc5/6 still use HEAT proteins to generate a transient ATP-binding-dependent gripping state on duplex DNA en route to topological loading? At the same time, structural studies provide an architectural understanding of the DNA replication machinery, which in turn can help address the structural mechanism of sister chromatid cohesion. By physically mapping cohesin conversion factors towards the front of the advancing replication machinery and de novo loading factors at the rear of the helicase, we can start building a structural framework for cohesion establishment at the replication fork.

Cohesin conversion in cells may require cohesin to change its conformation in order to be passed by the replication machinery. The CMG helicase measures ~23 nm along its largest dimension [42], while the extended cohesin ring measures ~30–40 nm in length [112]. As discussed above, DNA-roadblock passage experiments indicate that the pore diameter of loaded cohesin ranges between ~10.6 and ~19.5 nm [105], suggestive of a collapsed ring conformation. This observation makes the prediction that cohesin may thus be pushed along DNA by the translocating helicase. Passage of cohesin, on the other hand, would require conformational rearrangements within cohesin, which could be promoted by cohesion establishment factors. As a result, cohesin may adopt a wide ring configuration, which could be traversed by the replisome. The epistasis group for cohesin conversion [100,101], Tof1/Csm3, Ctf4, and Chl1, could play an important role in promoting this transition, as these factors all map at the leading edge of the advancing replisome. Alternatively, cohesin could dissociate from duplex DNA in front of the helicase and reassociate behind the replication fork without ever losing contact with the replication machinery. This conformational transition might be promoted by the capture of the single-stranded lagging-strand template [19,62,63]. Other questions about cohesion establishment remain to be addressed. For example, can loop-extruding cohesins be converted to cohesion, or does the replisome push the loop-extruding cohesin off DNA? How are cohesin-induced phase separation events resolved in living cells for the replisome to access DNA? Can the helicase translocate through cohesin clusters? Fluorescence imaging of single molecules in cells and in reconstituted systems, especially when combined with structural investigation, promises to address these questions.

Single-molecule imaging allows the visualisation of individual nucleic acid interactions with millisecond temporal resolution while monitoring the full context of a DNA substrate and a set of fluorescently labelled factors. While cryo-EM can routinely achieve near-atomic resolution for DNA-processing enzymes, frontier challenges for structural biology of chromosome maintenance are (i) describing nucleoprotein ultrastructures, such as the entire replication start sites containing loaded cohesin and helicase factors, and (ii) characterising the cascade of events as they unravel in reconstituted cellular pathways, such as the establishment of a replication fork and of sister chromatid cohesion. Improvements on multiple fronts can be leveraged. For example, cryo-EM approaches can be used to retrieve information on the relative orientation of distinct high-resolution macromolecular assemblies to reconstruct the full context of reconstituted DNA transactions. Repositioning averaged structures onto lower-signal input volumes is a routine approach for cryotomography of cells and viruses [113,114]. However, this strategy, named “RECONstitution in SILico” (RECONSIL), is only starting to be used in single-particle analysis of origin-dependent reactions. RECONSIL has so far been used to elucidate the mechanism of MCM DH loading onto chromatinised replication origins. Here, individual ORC molecules could be oriented with respect to nucleosomes that mark the origin boundaries, and reveal a sequential mechanism for the loading of two MCM rings to form the symmetric DH [1]. Applied to DNA entrapment by cohesin at the replication fork, this strategy will enable the establishment of whether cohesin loaded on parental DNA can be bypassed by the advancing replisome and whether the CMG can traverse the helicase ring or cohesin opening and closing is required in the process.

A second key development is time-resolved cryo-EM imaging, which has been implemented on the minute-resolution timescale to follow DH loading onto origins [1]. Such temporal resolution was sufficient to identify novel helicase loading intermediates that informed the mechanism of origin licensing; however, it might not be sufficient to capture ATPase-dependent events, such as cohesin bypass by the replisome. To circumvent this issue, millisecond resolution approaches to prepare cryo-EM grids have been developed, which employ microfluidic devices for sample mixing and incubation, combined with blot-free plunge freezing technologies [115,116,117]. These promise to be the future of structural enzymology and will likely enable us to study key short-lived intermediates in the sister chromatid cohesion reaction and other essential chromosome maintenance processes.

## Data Availability

Not applicable.
